# Modelling the Rhizosphere Priming Effect in Combination with Soil Food Webs to Quantify Interaction between Living Plant, Soil Biota and Soil Organic Matter

**DOI:** 10.3390/plants11192605

**Published:** 2022-10-03

**Authors:** Oleg Chertov, Yakov Kuzyakov, Irina Priputina, Pavel Frolov, Vladimir Shanin, Pavel Grabarnik

**Affiliations:** 1 Department of Natural Sciences, Bingen University of Applied Sciences, Berlin Str. 109, 55411 Bingen, Germany; 2 Department of Soil Science of Temperate Ecosystems, Department of Agricultural Soil Science, Georg-August-Universität Göttingen, Büsgenweg 2, 37077 Göttingen, Germany; 3 Agro-Technological Institute, RUDN University, 117198 Moscow, Russia; 4 Institute of Physicochemical and Biological Problems in Soil Science, Pushchino Scientific Center for Biological Research of the Russian Academy of Sciences, Institutskaya 2, 142290 Pushchino, Russia; 5 Center for Forest Ecology and Productivity of the Russian Academy of Sciences, Profsoyuznaya st., 84/32, bld. 14, 117997 Moscow, Russia

**Keywords:** priming effect modelling, root exudates, rhizosphere interactions, nitrogen mining, soil food web, available nitrogen

## Abstract

A model of rhizosphere priming effect under impact of root exudate input into rhizosphere soil was developed as an important process of the plant-soil interaction. The model was based on the concept of nitrogen (N) mining, compensating for the N scarcity in exudates for microbial growth by accelerating SOM mineralisation. In the model, N deficiency for microbial growth is covered (“mined”) by the increased SOM mineralisation depending on the C:N ratio of the soil and exudates. The new aspect in the model is a food web procedure, which calculates soil fauna feeding on microorganisms, the return of faunal by-products to SOM and mineral N production for root uptake. The model verification demonstrated similar magnitude of the priming effect in simulations as in the published experimental data. Model testing revealed high sensitivity of the simulation results to N content in exudates. Simulated CO_2_ emission from the priming can reach 10–40% of CO_2_ emission from the whole Ah horizon of boreal forest soil depending on root exudation rates. This modeling approach with including food web activity allows quantifying wider aspects of the priming effect functioning including ecologically important available N production.

## 1. Introduction

Crucial processes of plant-soil interactions in the rhizosphere (soil volume directly influenced by roots) are the priming effects [1,2,3]. The rhizosphere priming effect (hereinafter referred as PE or “the priming”) represents an increase of microbial activity by utilization of readily available organic substrates (root exudates and other rhizodeposits) released from living roots and leading mostly to acceleration of soil organic matter (SOM) decomposition [1,4,5]. This phenomenon takes place because of increases of extracellular enzyme production and activity by microorganisms [6,7,8,9] under impact of the exudates in a case of lack of nitrogen for microorganisms’ growth [10].

There is currently a wealth of experimental materials on the priming regarding its functions in the “plant-soil” system, and its role on a global scale [11,12]. The priming has also been discussed as a “destabilisation mechanism” for accelerating organic carbon (C) mineralisation and CO_2_ emission under climate change [13].

Root exudates (hereinafter “exudates”) represent a part of plant net primary productivity (NPP) and are a main component of rhizodeposition [14,15,16] which includes also mucus, epithelial cells and even dead fine roots. The exudation is dependent upon species-specific and environmental factors such as plant physiology and age, root surface area, density of root hairs, nutrients content in roots and rhizosphere soil, and atmospheric CO_2_ concentration. According to estimates [17,18,19], the total amount of exudates varies between 1.0 and 12.0% of the net fixed carbon (C), which is a notable part of the total C input in terrestrial ecosystems. Quantitative assessment of the organic C flows in the system of “exudates-microorganisms-rhizosphere SOM” is evaluated by ^13^C or ^14^C labelling and tracing, mainly under controlled conditions with annual plants or tree seedlings [18,20,21]. Studies focused on capturing and direct measurements of exudation from forest trees in situ are rare [19,22].

The input of readily assimilated organic substances (such as free sugars, amino acids, or organic acids) in the rhizosphere triggers soil biota for an increase of SOM mineralisation and acceleration of nutrient release, notably nitrogen (N), available for plant roots [21,23,24]. The majority of these PE mechanisms are related to the modifications of microbial activities and functioning: extracellular enzyme synthesis leading to SOM mineralisation, acceleration of microbial metabolism, consumption of microorganisms by soil fauna, killing by phages, change of microbial community structure and activation of various microbial groups [25]. The existing concepts of PE mechanisms emphasise the activation of microbial metabolism to accelerate the SOM mineralisation to fill the N deficit in exudates for microbial growth and activity [26,27,28]. Nitrogen release by accelerating the SOM mineralisation (be termed “N mining”) is an important integrative mechanism of priming effects [29].

The priming also includes effects where the input of available organic matter into the soil leads to a reduction in SOM mineralisation [25]. This “negative” priming can be observed when N-rich exudates enter the rhizosphere soil with a high SOM and a low N content [4]. It also can take place in some specific cases when microorganisms utilise easily decomposable substrate, ignoring recalcitrant SOM [30].

All the above assumes that coupling between root exudates, C and N availability and SOM decomposition stimulated by PE has to be accounted in modelling C and N cycling in soils and terrestrial ecosystems, especially under climate or land-use changes. Incorporation of PE mechanisms into soil and ecosystem models is needed to precisely simulate trajectories of plant production and soil C storage under future conditions [31,32].

The mathematical modelling is a powerful tool for science-based prediction of biological systems’ behaviour, and first attempt for the priming modelling was performed more than 40 years ago [33] with a general accent on the role of microbial activity. Molina et al. [34] performed simulation of N mobilization at priming to be integrated into the structure of SOM dynamic model NCSOIL. The linkage of the priming to SOM dynamics models was realised by Fontaine and Barot [35] with two functional groups of microbial communities (r and K strategists) as main drivers of CO_2_ emission at priming.

A more detailed priming model by Blagodatsky et al. [10] was based on a set of laboratory experiments with ^13^C and ^14^C labelling. The model quantifies the soil microbial activity with the following calculation of C fluxes. Nitrogen was considered in the model as a factor influencing microbial growth and CO_2_ emission. This model well reproduced priming under optimal conditions for soil biota. The ORCHIMIC model [36] develops this approach with a well parametrised microbiological core, detailed consideration of all phases of SOM decomposition and PE calculation.

A theoretical analysis of possibilities to incorporate priming in SOM models was performed by Wutzler and Reichstein [37], who proposed and preliminary tested three structural modifications of SOM dynamic models with priming functional feedback. However, their promising approach was not further developed. A simple PE modelling procedure was realised by Cheng et al. [1]. They used a version of the Century model, PhotoCent [38], and increased decay rates of the active (microbial), slow and passive SOM pools by 10%, 30% and 50%, respectively, to simulate the priming effect. Later, Perveen et al. [32] realised a more comprehensive dynamic approach by compiling a model with incorporated priming effect procedure based on the approach of Fontaine and Barot [35]. The model represents a “plant-soil” system and has two functional groups of microorganisms.

There are also models of the priming for specific sites and for rhizosphere processes related to exudate dynamics [39,40]. Finzi et al. [41] used a combination of two microbial decomposition models [42,43] to study exudate flux in the rhizosphere with the conclusion that about one third of CO_2_ emission in forest ecosystem is produced by priming. Bastida et al. [11] elaborated and used a “structural equation model” (SEM) as a set of statistical methods for processing data from 86 sites all around the world to find ecological predictors (factors) determining the soil priming. Mean annual temperature, climate aridity, soil C, P and clay contents, vegetation type, microbial biomass were used as predictors. It was found that soil C is the most important predictor of the priming on global level.

The priority of further development of priming modelling was delineated to quantifying the role of the priming in climate change [1,11]. Actually, recent priming studies and prospects of modelling are concentrated on C flux from soil only with low attention to ecosystem functions of the priming. The influence of priming on soil and ecosystem processes and the positive role of the priming in plant nutrition with N release from SOM is recognized [3]. However, the existing models consider N mining in the rhizosphere priming as a mechanism for replenishing N deficiency mainly for microbial growth [10,41]. The algorithm for calculating plant-available N that can be an outcome of PE processes is, to our best knowledge, not implemented yet in the SOM models.

This study is aimed to develop a model of the rhizosphere priming induced by root exudate input into N-limited soil, which is a typical case for the most of natural terrestrial ecosystems in temperate and boreal zones [44,45]. It is necessary to point out that it is exclusively the positive priming effect. We follow the widely used approach of SOM modelling [13,34,46] and soil food web [47,48] that considers the soil carbon dynamics (dC/dt = *k*_i_C). Using this approach, we experimentally assessed rates of soil C changes (*k*_i_) trace paths of soil and biota C transformation and integrated all biochemical phases of SOM decomposition. We propose a model which allows specifying carbon dynamics and tracking the path of nitrogen from exudates and SOM to microbial growth and back to roots. It was done by the inclusion into the model a faunal food web procedure from the Romul_Hum model [46,49]. It is a new aspect in the PE modelling that allows coupling microbial and faunal role in C and especially N pathways in the rhizosphere. The developed model can be a module linked with the SOM and terrestrial ecosystem models, where this pattern of plant-soil interactions is still underestimated [1,20,46,50]. This module will be integrated into the Romul_Hum model [49] to calculate the priming-induced CO_2_ emission and available N. The model also allows assessing the role of the exudate-induced priming to produce SOM and available N in food webs of soil biota.

## 2. Results

### 2.1. Model Description

The model describes processes of the positive rhizosphere priming [25] where microorganisms (MO) consume the released root exudates (RE) within 1–5 days [51] but with a rather long-term effect on C dynamics. Rapid microbial consumption of RE precludes any back re-assimilation of organic N by the roots [52]. However, microbial growth due to the exudates’ dissolved organic matter can be limited by the lack of N in this easily available organic substrate [53]. In this case additional nitrogen for MO growth is taken from the rhizosphere SOM by a specific process of its mining by soil MO [29,54,55] that means some increase of SOM decomposition. The model also has a built-in food web procedure for calculating consumption of MO by soil microfauna (“microbial grazers”) with the release of products of faunal metabolism and specifically mineral N, which can be used by plants [48,56,57,58].

**Figure 1:**
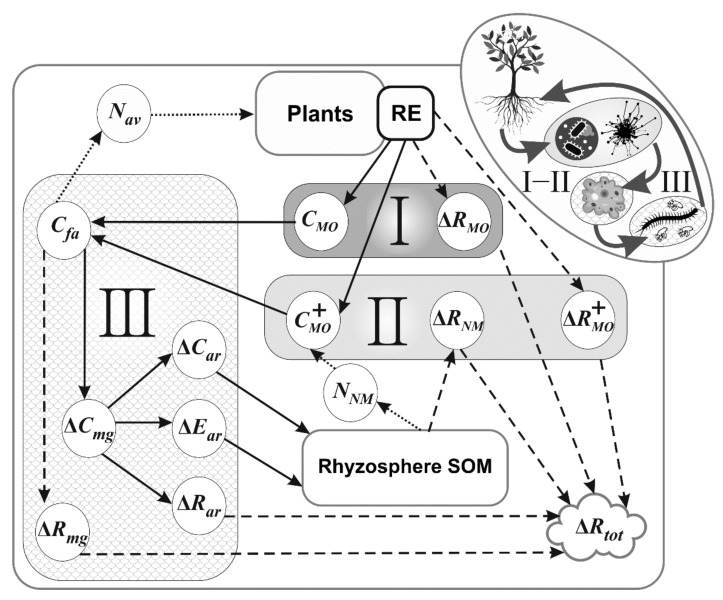
Conceptual structure of the rhizosphere priming model. “I” is a subroutine of root exudate (RE) consumption by microorganisms; “II” is a subroutine of nitrogen mining, and “III” refers to the soil fauna food web. The symbols are the same as in the equations in the “Model description”: “*C_i_*” is carbon of microorganisms and fauna biomass; “*R_i_*” relates to the respiration of soil biota; “*N_i_”* is nitrogen; “*E*” is faunal excrements. Symbol *∆* relates to the rate of the processes. Subscripts in symbols: “*MO*”—microorganisms; *“NM”*—nitrogen mining; “*fa*”—food for fauna; “*mg*”—microbial grazers; “*ar”*—arthropods; “*tot”*—total. $\Delta R_{MO}^{+}$ is a microbial respiration at the consumption of excessive C of exudates. The growth of microbial biomass owing to root exudation and change of food web components are represented with solid lines, the microbial and faunal respiration with dashed lines, the nitrogen mining and N release processes with dotted line. All is in carbon (C) or nitrogen (N) mass units. The formation and flow of microbial necromass to the rhizosphere SOM is not represented here. Input and output variables of the model are represented in Table 1. The model has a daily time step. All flows (denoted with *Δ* in the equations below) are calculated first, and after that the pools are changed at a single step. The parameters of the model are represented in Table 2.

This model is compiled for incorporation into the EFIMOD—Romul_Hum model system [46,59], in which the amount of root exudates will be defined as a proportion of plant GPP [60]. The structure of the model is represented on Figure 1. The model consists of three subroutines: (i) microbial growth due to the available C and N of exudates; (ii) microbial growth due to using residual C of exudates and N being extracted from the rhizosphere SOM (termed “N mining”); (iii) a formation of faunal by-products (excrements and necromass) and available N (ammonium) by soil fauna grazing on microorganisms within soil food web.

#### 2.1.1. Microbial Growth Caused by the C and N of Root Exudates

Exudates entering the rhizosphere soil from fine roots contain significant amounts of organic C: the primary assimilates of photosynthesis (glucose), carbonic acids, polysaccharides and amino acids [23]. The composition of exudates in the model testing was set by 15% amino acids as a mean from published data [62,63]. It corresponds to 1.25% N in the whole material (C:N ratio is 40).

Soil of the rhizosphere has definite pools of organic C, N and soil biota biomass. The initial pool of rhizosphere MO biomass was accounted as a function of rhizosphere SOM [49]. For algorithm description, we denote C and N content in root exudates daily flow as $\Delta C_{RE}$ and $\Delta N_{RE}$, respectively. We assume all exudates’ N and part of exudates C are fast consumed by the microorganisms (for a day or any other time step Δt) and used for their biomass growth (C and N) and respiration (only C). Other parts of exudates’ C, $\Delta C_{MO}$, can be consumed by the microorganisms using additional sources of available N [6,9]. This means that our starting point of the proposed model is based on the relations$\;\Delta C_{MO}<\Delta C_{RE}$ and $\Delta N_{MO}=\Delta N_{RE}$.

The single step of the model describes the change of the C pool of microbial biomass due to RE consumption, $C_{MO}\left(t\right),$ as a function of time t simply as

(1) $$C_{MO}\left(t+\Delta t\right)=C_{MO}\left(t\right)\times \left(1-K_{f}\right)+\Delta C_{MO}\times K_{AS\;},$$

where $K_{f}$ is a rate of microbial grazers’ feeding on MO; $K_{AS}$ is a rate of RE assimilation by MO. In turn, the microbial biomass daily increment, $\Delta C_{MO},$ driven by the available N in exudates, is calculated as follows:

(2) $$\Delta C_{MO}=CN_{MO}\times \Delta N_{RE},$$

where $CN_{MO}$ is the C:N ratio of the microbial community which is dependent on the rhizosphere soil C:N ratio ($CN_{SOM}$ in the Equation (3)) and is calculated with the modified function by Chertov et al. [49]:

(3) $$CN_{MO}=CN_{b}+\frac{\left(CN_{f}-CN_{b}\right)}{\left(1+\exp\left(-0.49\times \left(CN_{SOM}-13.23\right)\right)\right)},$$

where $CN_{b}$ is a C:N ratio of bacterial community (by default set as 5) and $CN_{f}$ is a C:N ratio of fungal community (by default set as 14). The $CN_{MO}\;$is also used in some equations below.

A substantial part of the consumed exudates is used for respiration at the microbial growth, $\Delta R_{MO}$, and is calculated as follows:

(4) $$\Delta R_{MO}=\Delta C_{MO}\times \frac{1-K_{eff}}{K_{eff}},$$

where $K_{eff}$ is the factor of MO growth efficiency from assimilated exudates carbon. It is assumed in the model that the exudate C is fully assimilated by microorganisms. The $K_{eff}$ = 0.3, which was originally used in the model [48] is lower than the measured values of the “carbon use efficiency” (CUE in microbiology, [64,65]) showing that it is a varying parameter. Therefore, we calculated and adopted a function of $K_{eff}$ depending on C:N of root exudates, $CN_{RE}$, and C:N of microorganisms, $CN_{MO}$, using data by Manzoni et al. [66]:

(5) $$K_{eff}=5.005\times {\left(\frac{CN_{RE}}{CN_{MO}}+5.276\right)}^{-1.038},R^{2}=0.56$$

#### 2.1.2. Microbial Growth Due to Using Excessive C of Exudates and Mined N from the Rhizosphere SOM

We postulate that the C part of exudates cannot be utilized for microbial growth when all N in exudate was fully used. This excessive C ($\Delta C_{RE}^{rest}$) is calculated as:

(6) $$\Delta C_{RE}^{rest}=\Delta C_{RE}-\Delta C_{MO}-\Delta R_{MO}.$$

We postulate that this excessive C is fully used for MO growth owing the process of N mining from rhizosphere SOM. The increment of microbial biomass due to the excessive C of root exudates, $\Delta C_{MO}^{+}$, is calculated as follows:

(7) $$\;\Delta C_{MO}^{+}=\Delta C_{RE}^{rest}\times K_{eff},\;$$

where $K_{eff}$ is described above.

Bacteria and fungi first use sugars and amino acids from the exudates and therefore, the residual C of exudates is represented mostly by various organic acids. “Excessive” C of exudates and “new” microbial biomass plays a role of the microbial enzyme system activator resulting in accelerating the mineralisation of the rhizosphere SOM [27], and thus the N release, that is termed “N mining” [29]. It can be assumed that the acid nature of the rest of the exudates also contributes to N mining by a depolymerisation of recalcitrant soil carbon with N release.

The dynamics of excessive carbon of exudates $C_{RE}^{rest}\left(t\right)$ is described as follows:

(8) $$C_{RE}^{rest}\left(t+\Delta t\right)=C_{RE}^{rest}\left(t\right)+\Delta C_{RE}^{rest}-\Delta C_{MO}^{+}{}_{\;}-\Delta R_{MO}^{+},$$

where $\Delta C_{MO}^{+}$ is C of microorganism growth due to the excessive C when additional N is mined by accelerating SOM mineralization and which contributes in dynamics its pool $C_{MO}^{+}\left(t\right)$:

(9) $$C_{MO}^{+}\left(t+\Delta t\right)=C_{MO}^{+}\left(t\right)\times {\left(1-K_{f}\right)}_{\;}+\Delta C_{MO}^{+},$$

where $K_{f}$ is the faunal assimilation efficiency as explained in Equation (1).

In Equation (7) $\Delta C_{MO}^{+}$ is the C of microbial growth which takes place due to consumption of excessive C of exudates by the microorganisms. Microbial respiration due to this process, $\Delta R_{MO}^{+}$, is calculated as follows:

(10) $$\Delta R_{MO}^{+}=\Delta C_{RE}^{rest}\times \left(1-K_{eff}\right),$$

where all variables are described above.

The N mining, $N_{NM}$, is N required for the use of excessive exudates for the microbial growth which is “mined” from the rhizosphere SOM. In the model it is depended on the microbial C:N, $CN_{MO}$, by the relation:

(11) $$N_{NM}=\frac{C_{MO}^{+}}{CN_{MO}},$$

where all variables are as described above.

In the process of N mining, the rhizosphere SOM mineralization takes place with a total C-CO_2_ emission, $R_{NM}$, being calculated in dependence on SOM C:N ratio:

(12) $$R_{NM}=N_{NM}\times CN_{SOM}.$$

This $R_{NM}$ is equal to soil C pool that was mineralised with a rate of RE assimilation by MO, *K_AS_* (Table 2). This flux of C-CO_2_ emission related to microbial respiration during N mining corresponds to the term “the real priming effect” [25] or “primed carbon” [67].

Synchronously with the described above priming processes being initiated by RE input, the rhizosphere SOM is also mineralised with C-CO_2_ emission that is calculated by Romul_Hum model [46].

#### 2.1.3. Food Web Processes of the Soil Faunal By-Products and Available N Formation

Calculated microbial biomass enters (a) the faunal food web (FW) according to the approach described by Chertov et al. [49], and (b) the rhizosphere SOM as a MO necromass [68]. In FW procedure, microorganisms are consumed by the “microbial grazers” (protozoans, nematodes and microarthropods) with ammonium release which is the N source for root uptake [48,56,57]. These are very fast processes of C and N turnover [69,70]. This trophic level serves as a food for the upper FW levels, where microbial grazers are consumed by soil micro- and mesofauna (mostly arthropods) with the formation of excrements and necromass, which are returning to the rhizosphere SOM as it was already performed in the Romul_Hum model [49].

Total C of MO assimilated by soil fauna, $\Delta C_{fa}$, at the first food web level of “microbial grazers” is calculated as follows:

(13) $$\Delta C_{fa}=\left(C_{MO}\left(t\right)+C_{MO}^{+}\left(t\right)+\Delta C_{MO}+\Delta C_{MO}^{+}\right)\times K_{f}\times \left(1-K_{MOm}\right),$$

where $K_{MOm}$ is MO mortality with necromass formation; other variables are described above.

In turn, the microbial grazers’ biomass increment, $\Delta C_{MG},$ is calculated as follows:

(14) $$\Delta C_{MG}=\Delta C_{fa}\times K_{MG},$$

where $K_{MG}\;$is the coefficient of assimilation of food by microbial grazers (production efficiency, taken equal to 0.4 [48]). The respiration of microbial grazers, $\Delta R_{MG}$, is calculated as follows:

(15) $$\Delta R_{MG}=\Delta C_{fa}\times \left(1-K_{MG}\right).$$

The increment of the biomass C of the next trophic level of the food web (arthropods) due to the consumption of microbial grazers,$\;\Delta C_{ar}$, is calculated as follows:

(16) $$\Delta C_{ar}=\Delta C_{MG}\times K_{ar}\times K_{arF},\;$$

where $K_{ar},$ is the coefficient of assimilation of food by arthropods (taken equal to 0.4 [48]);$\;K_{arF}\;$is microarthropods’ feeding rate.

The increment of the carbon of arthropod excrements, $\Delta E_{ar}$, is calculated as follows:

(17) $$\Delta E_{ar}=\Delta C_{MG}\times K_{arE},$$

where $K_{arE}$ is the coefficient that determines excreted portion of consumed food (taken equal to 0.2 [48]). This faunal by-product represents a feedback path from PE processes (including FW functioning) to the rhizosphere soil. 

The cumulative necromass of MO and arthropods, $\Delta C_{necr}$, can be expressed as:

(18) $$\Delta C_{necr}=\Delta C_{ar}\times K_{ARm}+\left(C_{MO}\left(t\right)+C_{MO}^{+}\left(t\right)+\Delta C_{MO}+\Delta C_{MO}^{+}\right)\times K_{MOm},$$

where $K_{ARm}$ is a rate of formation of necromass of arthropods, all other variables are described above. 

The C-CO_2_ flux due to respiration of arthropods, $\Delta R_{ar}$, is calculated as follows:

(19) $$\Delta C_{ar}=\Delta C_{MG}\times K_{ar}\times K_{arF},\;$$

where all variables are described above.

Summing it up, the total C-CO_2_ emission initiated by RE input to the rhizosphere is calculated as:

(20) $$\Delta R_{tot}=\Delta R_{MO}+\Delta R_{MO}^{+}+\Delta R_{NM}+\Delta R_{MG}+\Delta R_{ar},\;\;$$

where $\Delta R_{MO}+\Delta R_{MO}^{+}$ represents microbial respiration due to RE consumption, $\Delta R_{NM}$ relates to SOM mineralisation at N mining, and $\Delta R_{MG}+\Delta R_{ar}$ corresponds to the respiration of food web.

The cumulative rhizosphere priming for a given time interval is calculated as:

(21) $$R_{PE}=\underset{i}{\sum }\left(\Delta R_{tot}\right).$$

Then we calculated the sum of organic carbon, $C_{RS}$, returned to rhizosphere SOM with (both microbial and arthropods) $\Delta C_{necr}$, and faunal excrements $\Delta E_{ar}$, that partly compensates SOM losses because of its mineralisation due to N mining:

(22) $$C_{RS}=\underset{i}{\sum }\Delta C_{necr}+\Delta E_{ar}.$$

The production of ammonia as liquid excreta, $N_{av}$, at feeding of microbial grazers on microorganisms is calculated using function by Holtkamp et al. [48]:

(23) $$N_{av}=\Delta C_{fa}\times K_{f}\times \left(\frac{1}{CN_{MO}}-\frac{K_{MG}}{CN_{MG}}\right),\;\;$$

where $\Delta C_{fa}$ is the amount of carbon consumed by microfauna feeding on MO (Equation (13)) with $CN_{MO}\;$and $CN_{MG}$ being the C:N ratios of MO and microbial grazers, respectively. This excessive N-NH_4_ is calculated as the difference of MO biomass C:N ratio (5 to 10) and C:N of microbial grazers (10 to 12) taking also into account that 2/3 of MO carbon as faunal food is spending for the microbial grazers’ respiration [46,48].

### 2.2. Model Verification

**Figure 2:**
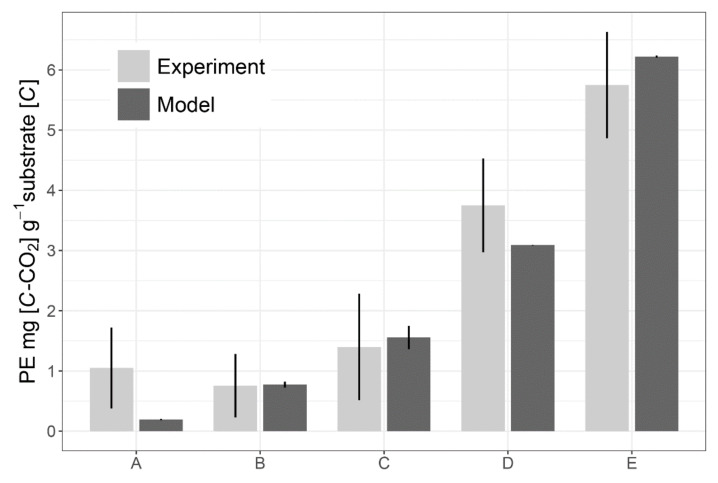
Comparison of the results of the priming effect (PE) measured in the laboratory experiment for subtropical forest soil [4] with simulated in the model and calculated as a sum of microbial respiration and rhizosphere SOM mineralization at nitrogen mining ($\Delta R_{MO}+\Delta R_{MO}^{+}+\Delta R_{NM}$). The initial data on C and N inputs with root exudates (RE) for model runs were identical to the data used in the experiment and imitated the following RE rates and their C:N: A—3 mg [C] g^–1^ soil, C:N = 20; B—12 mg [C] g^–1^ soil, C:N = 20; C—12 mg [C] g^–1^ soil, C:N = 80; D—48 mg [C] g^–1^ soil, C:N = 20; E—48 mg [C] g^–1^ soil, C:N = 80. The bars represent standard deviation. Simulation span is 30 days.

The model demonstrated the same order of PE intensities as in the experiment [4,10]. However, full consistency of the simulation results with experimental data was not observed. In the case of full N mining efficiency, the model overestimates the experimental results in the data set on O and Ah horizons of the forest soil [4] by 1.1 to 1.3 times in the case of high C:N value of exudates (C:N = 80) but without statistically significant difference between experimental and modelled data. (Figure 2). The model underestimates PE in comparison with this set of experimental data at low amounts and low C:N value of exudates (C:N = 20). The model overestimates, by up to 1.2 times, the experimental results in the data set on agricultural soil with low C:N values of soil (C:N = 12) and exudates (C:N = 10) (the data are not presented in Figure 2). The results of the model runs showed some peculiarities of the model behaviour. The PE clearly depends on the N mining to make up for its deficit for microbial growth, which is higher with increasing amounts of exudates and their C:N value. 

### 2.3. Analysis of Model Sensitivity to Parameter Variations

The analysis showed that the output data of the model are the most sensitive to the C:N ratio of microorganisms and, to a lesser extent, C:N ratio of rhizosphere soil. The very high R^2^ (0.937) of the linear regression proves that the effect of parameters on outputs is generally linear (Table 3).

### 2.4. Model Testing at the Level of Rhizosphere Soil

**Figure 3:**
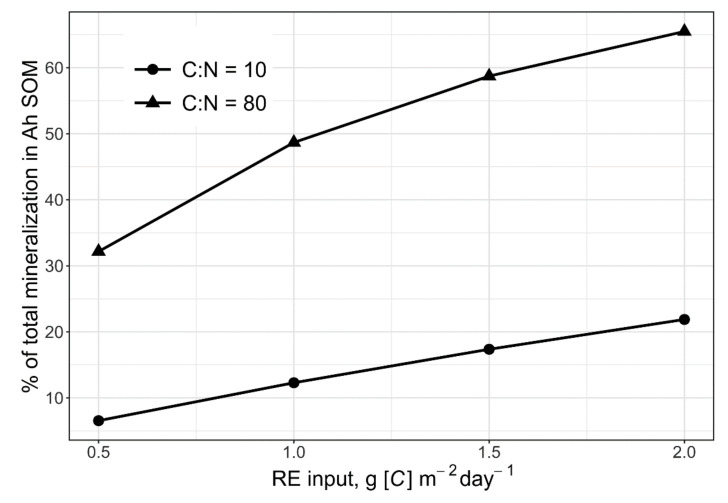
Simulated C-CO_2_ efflux at the priming depending on the exudates (RE) inputs and their C:N ratio as related to the bulk pool of mineralized C in a whole Ah horizon over 20 days. SOM pool in Ah horizon was 6.91 kg [C] m^−2^, the mineralisation rate of SOM was set at 0.00018 day^−1^ [46].

A set of model runs on the scale of a rhizosphere soil with the variation of exudate and microbial community parameters showed interesting results for the influence of several factors on PE. The simulation confirmed the conclusion of the model verification that the modelled PE was clearly dependent both on the amounts of exudates entering the soil and especially on their C:N ratios reflecting the exudate richness with N (Figure 3). In comparison with total SOM mineralisation in Ah horizon, the CO_2_ released by priming amounted for 10–20% for exudates with C:N = 10, and reached 30–65% when C:N was 80, i.e., the lower the N content in root exudates is (high C:N ratio), the higher is the PE.

**Figure 4:**
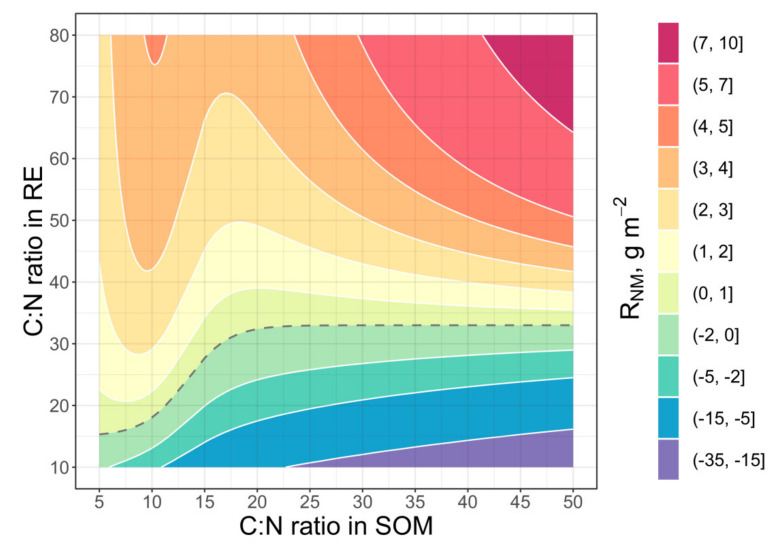
Simulated effect of extra C mineralisation at priming effect (*R_NM_* in Equation (12)) from rhizosphere SOM depending on C:N ratio of exudates and C:N ratio of the rhizosphere SOM. The simulation at the rhizosphere soil scale testing was run for 20 days; exudate input is equal to 0.5 g [C] m^–2^ day^–1^. An area with high C:N of RE and rhizosphere SOM has high PE while the area with very low C:N of RE has negative priming.

The PE simulation by the matrix [C:N ratio of RE] x [C:N ratio of rhizosphere soil] in a frame of really existent values (Figure 4) revealed a strong influence of these parameters on PE. A significant variation of its intensity was found in dependence on nitrogen status in the “root-soil” system. The high C:N ratio of RE (low N content) demonstrates a strong PE intensity that is largest one in poor soil with C:N ratio up to 50. Contrarily, low C:N of root exudates leads to diminishing of PE down to the negative values. The increasing of rhizosphere soil C:N (low N content) shows a more contrasting effect of nitrogen content in RE.

**Figure 5:**
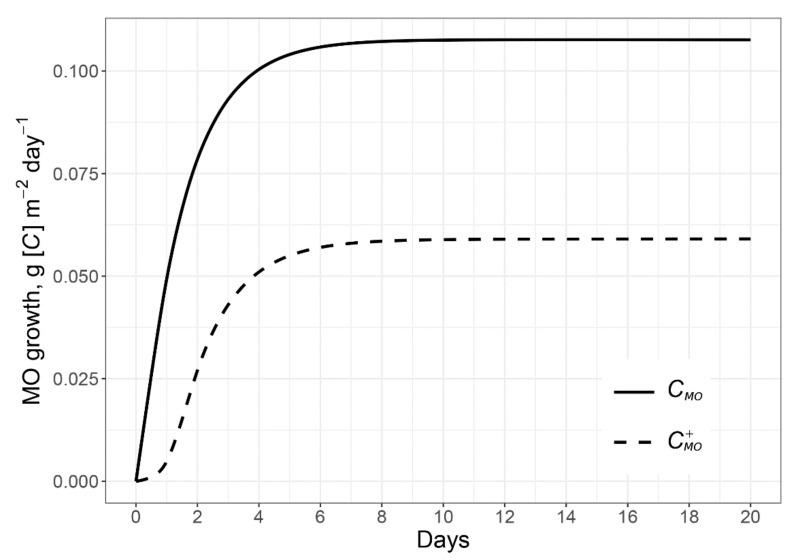
Simulated dynamics of microbial growth due to exudates (RE) input at the scale of rhizosphere soil. *C_MO_*—microbial biomass growth using C and N of exudates; $C_{MO}^{+}\;$–microbial biomass growth due to N mining for the microbial growth using the residual exudates C; RE input equals 0.5 g [C] m^−2^ day^−1^.

The model shows active growth of microorganisms in the rhizosphere due to both RE inputs and N mining (Figure 5). Simulated microbial biomass growth rates in the rhizosphere were significantly faster than in the root-free soil because of excess of available C in the rhizosphere and the nearly *steady state* conditions for microorganisms in the non-rhizosphere soil [71,72].

Model runs were also carried out with a range of microbial C:N ratios within the actual values for bacterial and fungal communities [73,74] which is dependent on the rhizosphere soil C:N ratio (Equation (3)). The priming and rhizosphere SOM mineralization due to N mining decreases if the microbial community has a high C:N ratio, which means domination by organisms with a low N demand (mainly fungi) and is typical for soils with high C:N ratio. A microbial community with low C:N (mainly bacteria) has a high N demand, possibly resulting in a larger PE if soil has a high C:N ratio. This gives data for preliminary evaluation of priming intensity in a wide range of edaphic conditions in boreal and temperate forests.

**Figure 6:**
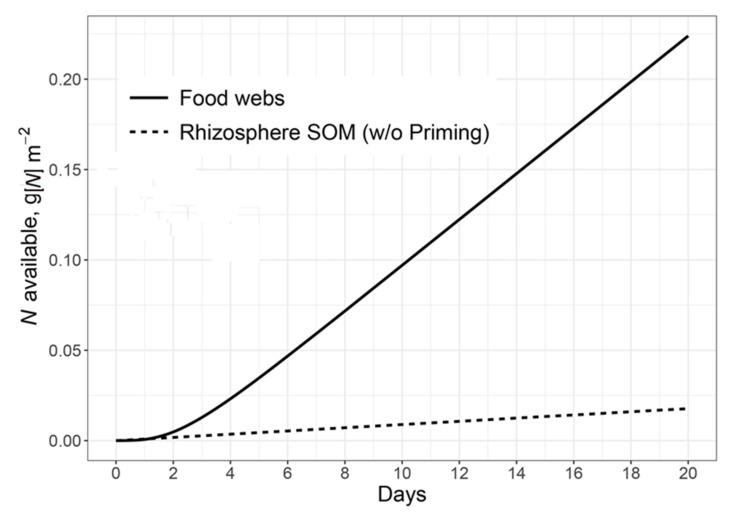
Cumulative N mineralization available for root uptake in the rhizosphere soil only. N is mineralized from two sources: available N produced in food webs (solid line) and mineralized N in rhizosphere SOM without exudate input (dotted line). C:N of root exudates is 40. The lag phase of the available N production within food webs is the effect of the daily time step of the model because microbial biomass needs to grow first before PE can be produced.

Finally, yet importantly, the amount of N available to plants (mainly ammonia) released within food webs is comparable with the N pool in the exudates and is in some times higher than the amount of N mineralised from SOM in the rhizosphere without priming effects (Figure 6). Thus, the sum of excessive N at the “microbial grazers” level of the food web as available for root uptake N plus excrements and necromass N can exceed the pool of N coming with exudates by 35%. This available N depends linearly on the exudate amounts and especially on their N pool.

Thus, mineralised N from the rhizosphere SOM (N mining) is used by microorganisms for growth, followed by the feeding of fauna on microorganisms in food webs with the release of excessive N at the level of “microbial grazers” and production of N-rich excrements and necromass [48,75].

### 2.5. Model Testing at the Level of Whole Soil Horizon

**Figure 7:**
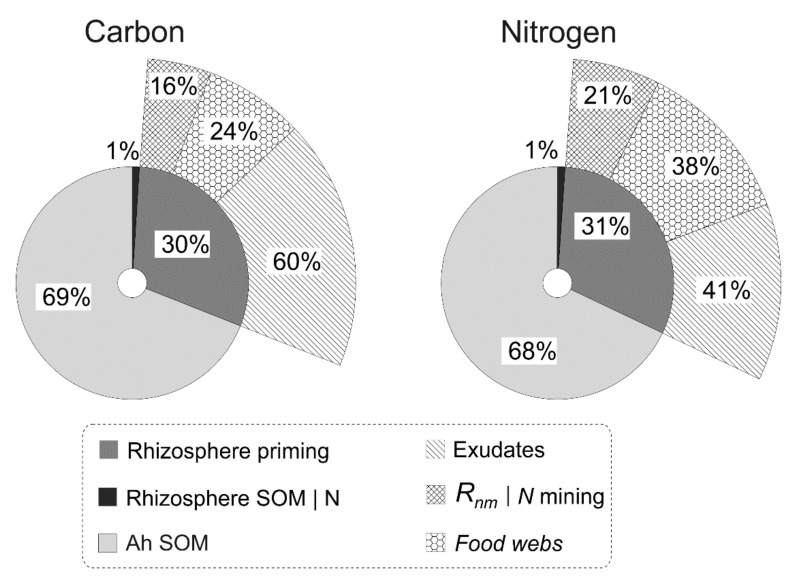
Simulated carbon (C) and nitrogen (N) fluxes in the forest soil (Ah horizon, pools of soil C and N are 6.91 and 0.49 kg m^−2^, respectively) with a regular root exudates input (0.5 g [C] m^−2^ day^−1^, C:N = 40) over 30 days. “Carbon” on the figure represents C-CO_2_ emission at priming (*R_PE_*, Equation (21)) plus C-CO_2_ of SOM mineralisation in the rhizosphere and bulk soil. “Nitrogen” is an available nitrogen mineralized in the rhizosphere and bulk soil of the whole Ah horizon plus nitrogen from root exudates mineralisation (*N_RE_*), produced by N mining (*N_av_*, Equation (22)) and obtained from faunal by-products. The sum of C and N fluxes (data used in circle diagrams) is 53.8 and 2.99 g m^−2^ month^−1^, respectively.

Model testing at the level of soil horizon accounts for both the processes in the rhizosphere and the entire organo-mineral horizon Ah of the forest soil (moder loamy Retisol). The simulated data on processes occurring in the rhizosphere with only 1.3% C stock of the whole Ah horizon are comparable in magnitude with data across the entire Ah horizon. Depending on the rate of exudates flow (3 or 15 g [C] m^−2^ month^−1^), the CO_2_ released by priming was 16 (at maximum) and 4.2 g [C] m^−2^ month^−1^ (at minimum) that is comparable with the CO_2_ emission from the entire Ah horizon (37.0 g [C] m^−2^ month^−1^). Mineralised C from the rhizosphere SOM at N mining (*R_NM_*) amounts for 16–28% of the released exudate C (Figure 7). At the maximum exudation, the amount of N mineralised (mined) from the rhizosphere SOM (0.19 g [N] m^−2^ month^−1^) and N produced by food webs during priming (0.35 g [N] m^−2^ month^−1^) is comparable with N released from root exudates (0.38 g [N] m^−2^ month^−1^). The same dependence is retained for the smaller RE pool. Depending on the rate of exudates release, the sum of N fluxes involved in the rhizosphere priming varies between 9–44% of the total pool of mineralised N during one month in the whole Ah horizon (2.05 g [N] m^−2^ month^−1^). These amounts can exceed 30 times the N mineralisation in the soil (0.027 g [N] m^−2^ month^−1^) without exudates input (Figure 7). This testing shows an expressive difference in the C and N flows structure and capacity at the priming functioning.

## 3. Discussion

This model was elaborated using a biogeochemical approach [13,76,77] where the dynamics of C and N are considered at the level of “plant-soil” interactions. In this approach, there is no necessity for reproduction of biochemical pathways in detail [78] as in the dynamic models of soil microbiological communities [1,10,36]. 

The general trend in research and existing priming models emphasises the dynamics of CO_2_ emission from soil as influenced by various factors [10,27,41]. The dominating approach in the PE modelling is a reproduction of the priming phenomenon similar to laboratory experiments with the main focus on C fluxes, microbial growth and activity [1,36,37], mostly ignoring positive ecological aspects of the priming. New insight on priming effect as a key process of C cycling in terrestrial ecosystems is now shifting the focus to the emission of excessive C to the atmosphere as a negative aspect of the priming affecting climate change [11]. The developed model accentuates a significant role of the priming in N release for plants and soil biota.

### 3.1. Priming Effect, N Mining and Food Webs as Processes of Fast Cycles of C and N in the Rhizosphere Soil

The approach proposed here is to model the most common type of the positive rhizosphere PE with a compensation of N limitation for microbial growth in the root exudates by the increase of soil C and N mineralization. The principal point in the model structure was to use the concept of N mining to meet the microbial demand for growth as a key factor of increased SOM mineralisation at the priming. The model allows for a quantitative assessment of general PE data on soil C dynamics (CO_2_ emission by mineralisation of exudates and SOM at N mining) that is a main aspect in the PE studies [3,10,11,27]. Generally, the results of model verification correspond to experimental data obtained under controlled conditions.

A specific feature of this simulation approach is the inclusion of a food web module into the model structure [48,49,61], which is absent in all current priming models. In the food webs, the basic level of soil fauna is a group of “microbial grazers” that consume microbial biomass. It consists of Protozoa, Nematoda and Microarthropoda [49,61]. This group, being the main consumer of microbial biomass, secretes into the soil the liquid excreta of mostly ammonia that is available N for plant roots [47,48,75]. This pathway of N release for plants and other biota was missed in all existing models of the priming effects. Incorporating food webs into the model allowed describing two feedback loops in the functioning of “roots-soil biota-SOM” system): one loop is for the pathway through MO and soil biota for releasing available N for plants and soil biota; another one is for the return of C with faunal necromass and excrements to the rhizosphere SOM. The active feedbacks from living plant to soil on the ecosystem scale are still not at the focus of current research. These are discussed globally and regionally [79,80]. This is a new aspect in the modelling the priming effect. The rate of the processes in these feedback loops is very high, and they were defined as a “terrestrial plankton” [69] or “fast cycles of biological turnover” [70]. This is the main difference between the approach implemented in the model and the existing concept of N mining for microbial growth and root uptake.

Including these processes into the model alters the perception of the priming as a source of CO_2_ surplus in the atmosphere only. The model results show that the N produced in priming is higher than the N arriving with exudates. Nitrogen release by priming is as important a role as N mining by ectomycorrhiza [81], and also helps N uptake by arbuscular mycorrhiza, which works mainly for plant stability and phosphorus supply [82].

### 3.2. Experimental and Simulated Data: Plant-Microorganism-Soil Fauna Interactions in the Rhizosphere Soil

The verification demonstrated a small overestimation of simulated results in comparison with experimental data. It might be because the dried, ground and sieved soil samples were used in all laboratory experiments. In this case, structure and function of soil fauna could not be fully reproduced, and its role was underestimated [61]. Previously, nearly all PE experiments were done with a start pulse addition of glucose or exudates [3,4]. There are only a few priming effect studies with repeated or continuous addition of organic compounds [67,83] or plant growth with continuous labelling [84,85,86]. Similar to experimental data [67,83], the elaborated model demonstrated some differences in the PE intensity (data not shown). The sensitivity analysis demonstrates that the model is more sensitive to the parameters of edaphic status of soil and microbial community, which are reflected by their C:N ratio. Those are in correspondence with the model behaviour at rhizosphere and whole soil horizon testing.

Model testing showed a fast response of the simulated PE to the N content in exudates (C:N ratio). The lower the N content of the exudates (high C:N), the higher CO_2_ emission by priming is, which follows experimental data [4]. Accordingly, the less N in exudates, the higher is the N mining intensity. 

Model testing at the level of whole soil horizon reflects the situation with a complete exudate uptake and utilisation by microorganisms due to N of exudates and N mining, and complete microbial biomass consumption by soil microfauna. The simulation results quantitatively assess the contribution of PE to the total CO_2_ emission as an important process that can reach up to one third of the CO_2_ emission from the whole soil that also was experimentally confirmed [11,40]. The impact of PE on CO_2_ emission from soil and on N mineralisation was consimilar. However, N is the main limiting factor determining the trophic status of forest ecosystems in most soils in temperate and boreal forests.

So far, N dynamics has been considered as a significant process in the PE phenomenon, regulating CO_2_ emissions [3,5,10] but without quantitative assessment of the nitrogen mineralization dynamics and intensity. In contrast to the previous studies, N in this model is also a rate variable driving the transformation processes through the C:N ratio. This is a new aspect of PE modelling, which has provided an opportunity to understand the role of PE in terrestrial ecosystems. Based on the results of the model runs with a 20% increase in mineral N output to the soil as a whole, PE can be considered as a substantial mechanism for additional mineralisation of N stored in the SOM. This N becomes available for the root uptake and nutrition of vegetation, thus increasing its productivity and stability. This can be interpreted as a “target function” of the priming effect from the ecological point of view.

The model runs consider the situations of positive priming effect. In some cases, the addition of labile C and N with exudates leads to decrease of CO_2_ emission from rhizosphere soil. This is what is known as “negative priming”, which seems to be an odd term because all meanings of word “priming” have a positive connotation. The decrease of SOM decomposition and CO_2_ emission happens because of competition in microbial community at strong nutrients insufficiency [26,87]. The PE laboratory investigation of a wide range of soil samples in a triangle “leaf litter-woody debris-recalcitrant SOM” [4] has shown that C:N of exudates higher 150 and C:N of SOM higher 50 lead to “negative” priming effect. Testing of this model in more detail with close to realistic N content in RE and SOM (Figure 4) identified additionally “negative” PE in a case of very N-rich RE (C:N < 10) in soils of various trophic level. It means that “negative” priming in natural ecosystems can take place mostly in dead wood and in root mats, or peat of extra oligotrophic grasslands and peatlands. However, it possibly can be in very rich agricultural soils/substrates where soil N does not limit plant growth.

### 3.3. The Model Uncertainties and Future Development

There is still a lack of some data that are necessary for this modelling approach. (a) In this model, N mining is presented as a “black box” without parameters of its functioning. (b) In spite of exudates composition and functions being thoroughly investigated [88], the actual input of root exudates needs to be specified because it varies greatly [18]. According to Pausch et al. [89], annual production of exudates is 166 kg [C] ha^−1^ year^−1^, which corresponds to 0.0002 g [C] m^−^^2^ day^−1^ for a 3-month vegetation period. In our simulation, calculating the flow of exudates as 3% of the net primary productivity of the forest stand [58] resulted in a figure of 0.1 g [C] m^−^^2^ day^−^^1^. (c) Soil macrofauna respiration of the upper levels of the food web was not related to SOM mineralisation [48] and was not accounted. (d) Testing of the model at the soil horizon level was performed without accounting for fluctuations in soil temperature, moisture and the impact of disturbances that occur in the natural environment. Therefore, the results of the model runs can be considered as the potential maximum capacity of the positive rhizosphere PE.

In the context of further development of PE modelling, some uncertainties should be clarified when including PE model into the terrestrial ecosystem models. First, the exudate amounts delivered directly to mycorrhizal symbionts bypassing the soil is necessary to parametrize [86,90,91]. On the other hand, ecto- and arbuscular mycorrhiza are sources of liquid excreta and enzymes (e.g., chitinase) and easily decomposable N-rich organics (e.g., chitin) of necromass [92], and consequently, mycorrhiza support the PE functioning.

It should be noted that an important requirement for improving this PE model, which combines the microbiological and faunal components of soil biota, is to obtain experimental data on the N mining, the amount and composition of root exudates as well as mineral N production [93,94]. 

## 4. Materials and Methods

### 4.1. Model Verification

Two sets of experimental data were used for the model verification. One set relates to forest soil in subtropical climate [4] (the Oa and Ah horizons of Alfisols, C_org_ = 42.5 and 10.5%, C:N = 19 and 16, respectively). Actually, there are 5 parallel experiments with different C:N values of exudates (Figure 2). The other set relates to agricultural soil in temperate climate [10] (the Ah horizon of Haplic Luvisol, C_org_ = 2.4%, C:N = 12). This data set has only 1 variant of the experiment. Both sets used the same experimental design. The initial data on input doses of labile C and N as root exudate mimics (C, N and C:N ratio) were identical to the values used in the experiment. The other parameters of the model used during verification was the same as presented in Table 2. It was one RE input at the first day of experiments and simulation. The simulation span was 30 days.

### 4.2. Sensitivity Analysis

Since the one-at-a-time approach is often criticized for its inability to account for the complexity brought by possible non-linear interactions of parameters, the analysis of the sensitivity of the model to the uncertainty in parameter estimation has been performed as proposed by Saltelli and Annoni [95]. According to this approach, for each input parameter a set of 11 values equally distributed in range of ±25% from the default value was generated, and then a dataset containing all possible combinations of the parameter values were compiled. The model was executed for one time step for each record in the dataset, where the priming effect (Δ*R_tot_*) was calculated. Generally, the approach consists in multiple linear regression analysis of standardized values $\left(\frac{\left(x_{i}-\bar{x}\right)}{S\left(x\right)}\right)$ of output variable with respect to the set of standardized values of input factors *I*_1_ … *I_n_*. Thus, the standardized regression coefficients *c*_0_ … *c_n_* (subscript 0 refers to an intercept) can be considered as the measure of sensitivity of the model to the certain parameter, while the value of R^2^ shows the non-linearity of the model (the lower the R^2^ the higher the non-linearity).

### 4.3. Model Testing

The model was tested on the data for Ah horizon of moder loamy Retisol from the mixed forest stand in the southern boreal zone of Eastern Europe [58]. The pools of C and N in the whole Ah horizon were estimated as 6910 and 490 g m^−2^, respectively. The stocks of C and N considered only in a small volume of rhizosphere soil around the fine roots of trees were 90.1 and 6.4 g m^−2^, respectively. The tree stand productivity (NPP) was estimated as 375 g [C] m^−2^ year^−1^. The average growing season duration was 225 days. The simulation was run for conditions of midsummer. The soil temperature was set at 16 °C with optimal soil moisture. The rate of microbial biomass consumption by soil fauna of food webs was estimated at 0.15 day^−1^ for bacteria and 0.06 day^−1^ for fungi [49,61,69]. The SOM mineralisation rate in bulk and rhizosphere soil were the same as in Section “Model description”. Other data and input parameters for model runs are presented in Table 2 and Table 4. Scenarios of exudate inputs to rhizosphere are described below.

#### 4.3.1. Rhizosphere Soil Level

The aim of model testing at rhizosphere level was to analyse the model behaviour depending on RE composition and quantity taking into account rhizosphere soil only. Model testing was performed with a set of runs by the matrix [exudate input] × [exudate C:N] with exudate input from 0.5 to 2.0 g [C] m^−2^ day^−1^ and for the C:N range from 10 to 80 reflecting its published values [63,67]. The simulation was run for 20 days with everyday exudate inputs.

#### 4.3.2. The Level of a Whole Soil Horizon

Testing at the whole Ah horizon level was aimed to estimate a possible contribution of the rhizosphere priming to total C-CO_2_ emission from main root layer of boreal soil. In the absence of measured data, two rates of root exudation were simulated. The first one was calculated based on the data for 25-years pine (*Pinus taeda*) plantation [19], where root exudate input from tree stand was estimated up to 3% of the NPP. Based on the NPP data for simulated tree stand from Chertov et al. [58], it corresponds to 0.1 g [C] m^−2^ day^−1^ (or 3 g [C] m^−2^ month^−1^). We considered this value as a minimal estimate for this soil since it did not take into account the root exudation from other forest vegetation. The second rate of exudation had a higher magnitude of 0.5 g [C] m^−2^ day^−1^ (or 15 g [C] m^−2^ month^−1^) shown for similar soil horizon in the experiment with 6-month coniferous seedlings [20]. The value of 0.5 g [C] m^−2^ day^−1^ was assumed to be a maximal estimate for the considered soil layer. The simulation was run for 30 days with everyday exudate inputs.

## 5. Conclusions

The proposed model of the rhizosphere priming effect is based on the concept of nitrogen mining from SOM combined with a soil food web module that is a new aspect in the priming simulation. It allowed for a quantification of a feedback in the “root-soil biota-SOM” system with releasing N for root uptake and returning some organic C back to soil organic matter. The N amounts released within the priming are similar and even higher than their amounts in the root exudates and so, are very relevant N sources for plant uptake. Therefore, the “target function” of the priming effect from the ecological point of view can be considered as the increase of SOM mineralization by microorganisms to mine N for plants and soil biota.

Testing the model with linkage to the entire Ah horizon revealed the high importance of priming at an ecosystem level: CO_2_ emission from the priming can reach up to 30–40% of CO_2_ released from the whole Ah horizon. The calculated production of mineral N at priming can be up to 1.5 times higher of the total N input with exudates because N mining and ammonium in liquid excreta of “microbial grazers”. This N excess justifies the root’s outflow of N to obtain its additional pool from the rhizosphere for increased plant growth and stability. Moreover, a returning of faunal by-products to the rhizosphere partially compensates intensive SOM mineralisation for N mining. The model parameters are supposed to be valid for soils in a boreal and temperate climate.

## Figures and Tables

**Table 1:** Model input and output variables.

Parameters	Measurement Units
Input variables	
Rhizosphere soil C	kg [C] m^−2^
Rhizosphere soil N	kg [N] m^−2^
Root exudate, RE, input	kg [C] m^−2^ day^−1^
Root exudate C:N ratio	-
$\mathrm{Microorganism}\;\mathrm{biomass},\;C_{MO}\left(t\right),$	kg [C] m^−2^
Microbial grazers biomass	kg [C] m^−2^
Arthropods biomass	kg [C] m^−2^
Output variables	
Total C-CO_2_ emission at priming	kg [C] m^−2^ day^−1^
C-CO_2_ emission at N mining	kg [C] m^−2^ day^−1^
C-CO_2_ emission at rhizosphere SOM mineralisation	kg [C] m^−2^ day^−1^
N available	kg [N] m^−2^ day^−1^
SOC, rhizosphere soil	kg [C] m^−2^
SON, rhizosphere soil	kg [N] m^−2^

**Table 2:** Parameters of the model.

Parameters and Source of Data in Square Brackets	Measurement Units	Amount/Value
Root exudates, RE, assimilation rate by MO, *K_AS_* [3,19,51]	day^−1^	0.50
Bacteria C:N ratio, *CN_b_* [48],	-	5.0
Fungi C:N ratio, *CN_f_* [48],	-	14.0
$\mathrm{Microbial}\;\mathrm{grazers}\;C:N\;\mathrm{ratio},\;CN_{MG}$ [48]	-	10.0
$\mathrm{Microarthropods}\;C:N\;\mathrm{ratio},\;CN_{ar}$ [48],	-	8.0
$\mathrm{Bacteria}\;\mathrm{and}\;\mathrm{Fungi}\;\mathrm{production}\;\mathrm{efficiency},\;K_{eff}$ [48]	-	0.30 *
Bacteria and Fungi respiration efficiency [48]	-	0.70
$\mathrm{MO}\;\mathrm{growth}\;\mathrm{rate}\;\mathrm{at}\;\mathrm{RE}\;\mathrm{input},\;\Delta C_{MO},$ [3,51]	day^−1^	0.50 *
SOM mineralisation rate [49]	day^−1^	0.00018
$\mathrm{MO}\;\mathrm{mortality},\;K_{MOm}$ [36,61]	day^−1^	0.04
$\mathrm{Grazers}\;\mathrm{feeding}\;\mathrm{rate}\;\mathrm{of}\;\mathrm{MO},\;K_{f}$ **	day^−1^	0.15
$\mathrm{Production}\;\mathrm{efficiency}\;\mathrm{of}\;\mathrm{microbial}\;\mathrm{grazers},\;K_{MG},$ [48]	-	0.40
$\mathrm{Production}\;\mathrm{efficiency}\;\mathrm{of}\;\mathrm{microarthropods}\;\mathrm{feeding}\;\mathrm{on}\;\mathrm{microbial}\;\mathrm{grazers},\;K_{ar}$ [48],	-	0.40
$\mathrm{Microarthropod}\;\mathrm{feeding}\;\mathrm{rate},\;K_{arF}$**	day^−1^	0.14
$\mathrm{Excreted}\;\mathrm{portion}\;\mathrm{of}\;\mathrm{consumed}\;\mathrm{food}\;\mathrm{by}\;\mathrm{microarthropods},\;K_{arE}$ [48]	-	0.20
$\mathrm{Microarthropod}\;\mathrm{mortality},\;K_{ARm}$ [48,61]	day^−1^	0.10

* initially used values; then they were calculated by Equations (5) and (7). ** authors’ evaluation using data by [48,61]. Note: The data of Kuzyakov [3], Holtkamp et al. [48], Phillips et al. [19], de Vries et al. [61], Chertov et al. [49], Huang et al. [36] and Liu et al. [51] were used. Production efficiency (food web terminology) corresponds to the ”carbon use efficiency” (CUE, microbiological terminology). Here and in tables below: the number of decimals depends on the source of data.

**Table 3:** Analysis of the model sensitivity to parameter variations.

Parameter	Parameter Name	Standardized Coefficient of Linear Regression
C:N ratio of soil	*CN_SOM_*	0.414 ***
C:N ratio of microorganisms (MO)	*CN_MO_*	−0.603 ***
C:N ratio of root exudates (RE)	*CN_RE_*	0.181 ***
Efficiency of RE assimilation by microorganisms	*K_eff_*	0.405 ***
Efficiency of MO assimilation by microbial grazers	*K_MG_*	−0.177 ***
Faunal assimilation efficiency	*K* *_fae_*	0.0534 ***
Intercept		~0
R^2^		0.937

Note: *** denotes the significance level (*p* < 0.001). The most influential parameter, ***CN_MO_***, is marked in bold. The *CN_RE_* parameter is used to calculate the N in root exudates based on C content. The $K_{fae}$ parameter is used in the food webs module [49].

**Table 4:** Parameters of test soil for simulation at the level of Ah horizon.

Parameters	Amount
Soil horizon Ah C pool, kg m^−2^	6.91
Soil horizon Ah N pool, kg m^−2^	0.49
Fine root specific length, m m^−2^	42.50
Fine root diameter, mm	1.50
Fine root dry weight, kg m^−2^	0.068
Diameter of rhizosphere soil tube (including root diameter), mm	7.50
Rhizosphere soil C pool, kg m^−2^	0.090
Rhizosphere soil N pool, kg m^−2^	0.0064
Root exudate input, kg [C] m^−2^ day^−1^	0.0001 … 0.0005

Note: the data of Chertov et al. [49] were used.

## Data Availability

The data are available from authors by reasonable request.
